# Prognostic value of pretreatment serum carbohydrate antigen 19-9 level in patients with colorectal cancer: A meta-analysis

**DOI:** 10.1371/journal.pone.0188139

**Published:** 2017-11-15

**Authors:** Zhan Yu, Zhen Chen, Jian Wu, Zhong Li, Yugang Wu

**Affiliations:** 1 Department of general surgery, The Third Affiliated Hospital of Soochow University, Changzhou, P.R. China; 2 Department of Urology, The Third Affiliated Hospital of Soochow University, Changzhou, P.R. China; 3 Department of general surgery, The second people's Hospital of Jiangyin, Jiangyin, P.R. China; University of Nebraska Medical Center, UNITED STATES

## Abstract

**Background:**

Carbohydrate antigen 19–9 (CA 19–9) is one of the most frequently used tumor markers for gastrointestinal cancer, particularly for diagnostic purposes. However, its value in predicting prognosis remains controversial. In this study, we sought to clarify this by conducting a meta-analysis of relevant studies.

**Methods:**

We systematically searched several databases, including PubMed, EMBASE and Web of Science for articles pertaining to the relationship between pretreatment serum CA 19–9 levels and prognosis in patients with colorectal cancer (CRC). The reported hazard ratios (HR) of overall survival (OS), disease-free survival (DFS), pooled progression-free survival (PFS) and recurrence-free survival (RFS) in the analyzed studies were compared by fixed effects/random effects models.

**Results:**

Seventeen studies involving 6434 patients with CRC were included in our meta-analysis. A comprehensive analysis of the collected data revealed that high serum CA 19–9 levels before treatment were significantly associated with poor OS (HR: 1.58, 95% CI: 1.36–1.83, *P*<0.001), DFS (HR: 1.71, 95% CI: 1.38–2.13, *P*<0.001), PFS (HR: 1.30,95%CI:0.93–1.82, *P* = 0.121) and RFS (HR: 1.43, 95% CI: 1.11–1.83, *P* = 0.006). This association between high pretreatment serum CA 19–9 levels and poor survival held true across different geographical regions, analysis types, methods used for HR determination, sample size, and treatment methods.

**Conclusions:**

The results of this study indicate that pretreatment serum CA 19–9 level can be used as a prognostic indicator for patients with CRC.

## 1. Introduction

Colorectal cancer (CRC) ranks third and second among the most common cancers detected in men and women, respectively, with an incidence of over 1.2 million and a mortality of 608,700 in 2008[1]. Over the years, considerable advances have been made in the treatment of CRC. Surgical resection still remains the mainstay in the treatment of patients with non-metastatic disease, but unfortunately, curative resection may not be possible at the time of diagnosis in most cases [2]. Therefore, the five-year survival rate for metastatic CRC remains poor[3]. Furthermore, there is still a lack of clarity regarding the optimal treatment for advanced CRC, the prognostic value of treatment, and effective prognostic markers.

Several screening modalities are currently available for colorectal cancer, including stool examination, colonoscopy, and computed tomography (CT). Many of these methods are invasive or have limited sensitivity and do not offer much value in prognostic evaluation. Nevertheless, the detection of cancer markers is a non-invasive method in the diagnosis of cancers. It is easily accepted by patients and is a simple procedure [4]. In clinical practice, tumor markers such as carcinoembryonic antigen (CEA) and carbohydrate antigen (CA) 19–9 are often used for the detection of adenocarcinomas [3,5,6]. Generally, serum CEA levels are elevated in the case of cancer recurrence, and therefore, this parameter is widely considered a marker for postoperative surveillance in CRC [7–9]. Although the specificity of CA 19–9 for detecting colorectal cancer is 96% [10], its sensitivity is only 23%, and its utility in predicting prognosis remains controversial [11]. In other words, elevated CA 19–9 levels have been reported to be strongly associated with poor prognosis in nodal-positive CRC after completion of adjuvant chemotherapy[12,13]. However, this method is not useful in predicting the prognosis in cases of nodal-negative CRC.

To date, there has been no systematic meta-analysis on the relationship between pretreatment serum CA 19–9 levels and the prognosis in patients with CRC. We aimed to overcome this gap in knowledge through this meta-analysis.

## 2. Methods

### 2.1. Search strategies

We conducted a systematic search of various databases, such as PubMed, EMBASE and Web of Science using the following search terms: “CRC,” “colorectal cancer,” “colorectal tumor,” “colorectal neoplasms,” “colon cancer,” or “rectal cancer;” and “survival,” “prognostic,” “prognosis,” or “outcome;” and “CA 19–9 antigen,” “gastrointestinal cancer antigen,” “CA 19–9,” or “carbohydrate antigen 19–9.” All entries meeting these criteria were manually retrieved.

### 2.2. Inclusion and exclusion criteria

Two investigators independently selected articles according to inclusion criteria. Disagreements were discussed with a third reviewer. Articles meeting the following criteria were included in the meta-analysis: (1) the diagnosis of CRC was confirmed by pathological examination; (2) data on OS, DFS, PFS, and/or RFS were provided to allow for the assessment of the relationship between serum CA19-9 levels before treatment and prognosis; (3) hazard ratio (HR) and 95% confidence interval (CI) values were directly provided or could be calculated. We excluded animal studies, editorials, reviews, comments, abstracts, meetings, or case reports.

### 2.3. Data extraction

The following data were extracted from the included studies: (1) study characteristics including the first author, country of origin, year of publication, number of patients, duration of follow-up, and method of survival analysis; (2) patient characteristics including geographical area, tumor site, age, gender, metastasis, treatment and cut-off value; (3) survival measures including HRs of OS, DFS, RFS, PFS and their 95% CIs. The HRs were extracted from multivariate or univariate analyses or estimated from Kaplan–Meier survival curves [14]. E-mails were also sent to the corresponding author to requesting the requested data.

### 2.4. Quality assessment

The quality of each study was assessed using the Newcastle–Ottawa Scale (NOS) [15]. If the score was more than 6, the study was considered to be of high quality.

### 2.5. Statistical analysis

From the data provided in each study, the HRs and their 95% CIs were calculated to assess the relationship between prognosis and pretreatment serum CA19-9 levels. We applied the random-effects model (DerSimonian–Laird method) in case of significant heterogeneity (*I*^*2*^≥50% and *P*<0.1) and the fixed-effects model (Mantel–Haenszel method) in the absence of heterogeneity. Data analysis was conducted using the Stata 12 Edition (Stata, College Station, TX, USA). Subgroup analysis was used to evaluate the data pertaining to geographical area, distant metastasis, the type of analysis, source of HRs, sample size, and the treatment method.

## 3. Results

### 3.1. Search result

**Fig 1:**
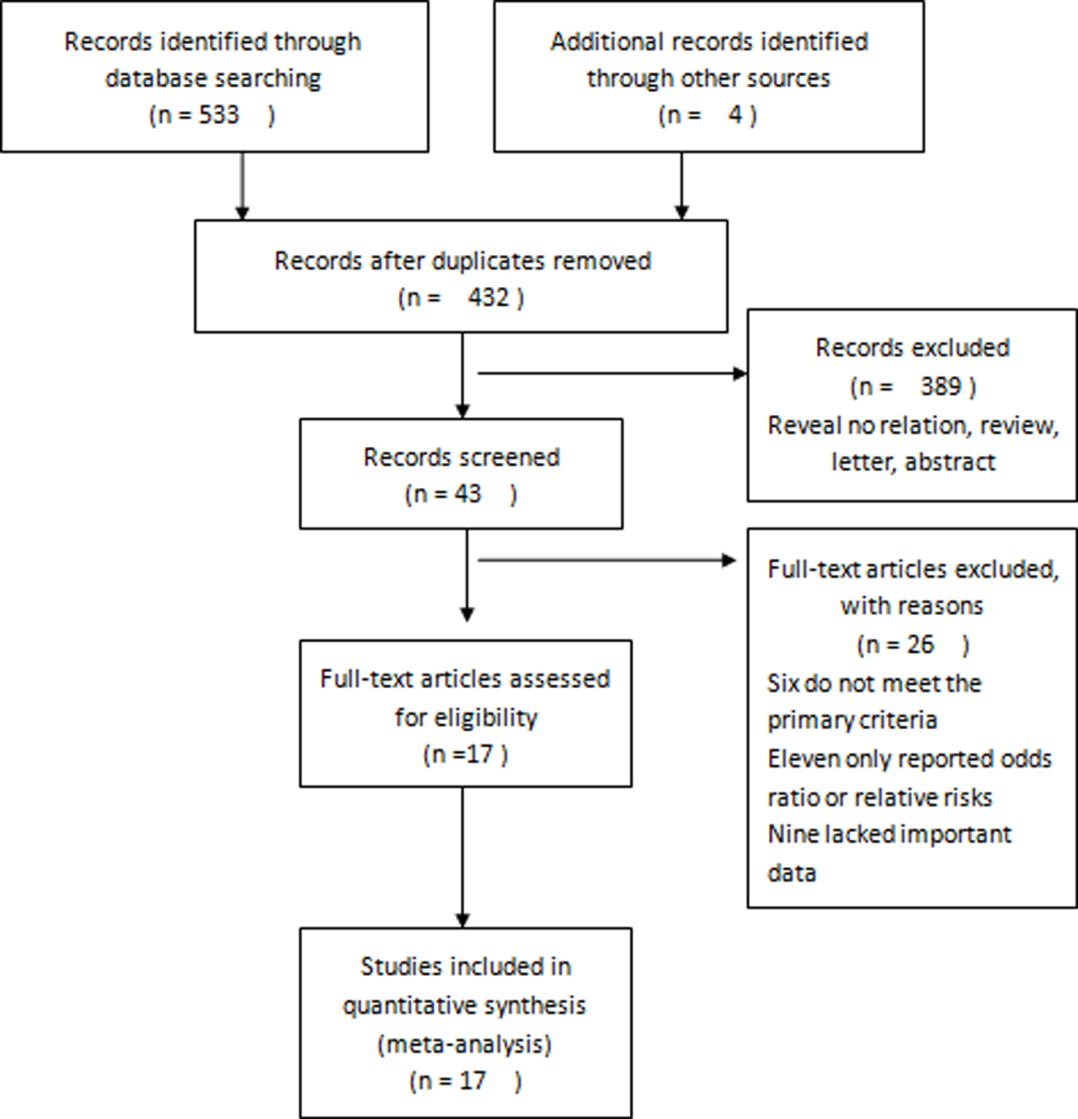
Flow diagram of the study selection process.

The systematic database search retrieved 537 articles. Each of these extracted articles was read, and 520 articles that did not meet the inclusion criteria were excluded from further analysis (Fig 1). Thus, 17 studies [11,16–31] comprising 6434 CRC patients were included in this meta-analysis, in order to assess the pretreatment levels of serum CA 19–9 as a prognostic biomarker in CRC.

The main characteristics of all 17 studies are summarized in Table 1. Fifteen of these studies provided data on the relationship between OS and pretreatment serum CA 19–9 levels. Five and two of these studies analyzed the relationship of pretreatment serum CA 19–9 level with DFS and with RFS and PFS, respectively. Among all 17 eligible articles, six studies were from China, six cohorts were from Japan, four studies were from the Korea, one study each was from Turkey and Czech Republic. HRs were recorded from the univariate analysis in four studies, determined from the multivariate analysis in 13 studies, and extracted from survival curves in two studies.

**Table 1:** Main characteristics of all studies included in the meta-analysis.

Author	Country/Year	Area	Tumor site	Case number	Age (years)	Gender (M/F)	Metastasis	Treatment	Follow-up (months)	Survival analysis	Cut-off value	Analysis	HR
Sookyung Lee	Korea /2016	Eastern	Colon/rectum 88/32	120	82/38 (<65/≥ 65)	59/61	No/yes 45/75	Mixed	median 7.6	OS	27	UV	report
Anna Song	Korea /2015	Eastern	Colon/rectum 125/52	177	123/54 (<65/≥ 65)	83/94	No/yes 69/108	Mixed	median 8.3	OS	27	UV	report
Mitsuru Ishizuka	Japan/2016	Eastern	Colon/rectum 418/209	627	169/458 (≤60/>60)	400/227	No/yes 491/136	surgery	median 29.9	OS	9.5	MV	report
Yuchen Wu	China/2016	Eastern	Colon/rectum 25/30	55	28/27 (<60/≥60)	35/20	No/yes 0/55	Mixed	NR	OS/PFS	37	NR	report
Yukiya Narita	Japan/2014	Eastern	Colon/rectum 148/104	252	Median 61	155/97	No/yes 0/252	chemotherapy	median 36.7	OS	37	MV	report
Jingtao Wang	China/2015	Eastern	Colon/rectum 176/134	310	138/172 (<65/≥ 65)	152/158	No/yes 310/0	surgery	median 71	OS/DFS	35	MV	report
Masatsune Shibutani	Japan/2015	Eastern	Colon/rectum 131/123	254	median 66	139/115	No/yes 254/0	surgery	median 1	OS	37	UV	report
Ondrej Fiala	Czech/2015	Western	Colon/rectum 86/66	152	median 61.1	104/48	No/yes 0/152	Mixed	median 18.9	OS/PFS	28	MV	report
Tsuyoshi Ozawa	Japan/2016	Eastern	Colon/rectum 96/77	173	mean 61	98/75	No/yes 0/173	surgery	median 36.9	OS/RFS	37	MV	report
Xian-Hua Gao	China/2013	Eastern	Colon/rectum 217/206	742	mean 60	423/319	No/yes 687/55	surgery	median 56	OS/DFS	37	MV	report
Z.-M Li	China/2016	Eastern	Colon/rectum 110/0	110	mean 62.9	58/52	No/yes 0/110	surgery	median 10.4	OS	37	MV	report
Ruixue Yuan	China/2013	Eastern	Colon/rectum/unspecified 184/182/5	371	mean 58.4	207/164	No/yes 341/30	surgery	mean 45.3	OS/DFS	37.5	MV	report
Fatih Selcukbiricik	Turkey/2013	Western	Colon/rectum 127/88	215	125/90 (≤60/>60)	133/82	No/yes 94/121	chemotherapy	median 30.8	OS	37	MV	report
HARUNOBU SATO	Japan/2011	Eastern	Colon/rectum 1476/0	1476	179/1296 (≤50/>50)	881/595	No/yes 1476/0	surgery	median Re/NRe 52.5/101.5	OS	37	MV	report
Shinya Abe	Japan/2016	Eastern	Colon/rectum 67/62	129	60/69 (<60/≥60)	80/49	No/yes 0/129	surgery	median 33.6	OS/RFS	50	MV	SC
IN JA PARK	Korea /2009	Eastern	Colon/rectum 534/575	1109	NR	614/501	No/yes 1109/0	surgery	median 48	DFS	37	MV	SC
Injae Hong	Korea /2015	Eastern	Colon/rectum 88/74	162	88/74 (≤62/>62)	90/72	No/yes 146/16	surgery	Mean 83	DFS	37	MV	report

Abbreviation: OS overall survival, DFS disease-free survival, PFS progression-free survival, RFS relapse-free survival, HR hazard ratio, NR not report, MV Multivariate analysis, UV univariate analysis, SC survival curve.

### 3.2. Meta-analysis

#### 3.2.1. Overall survival

**Fig 2:**
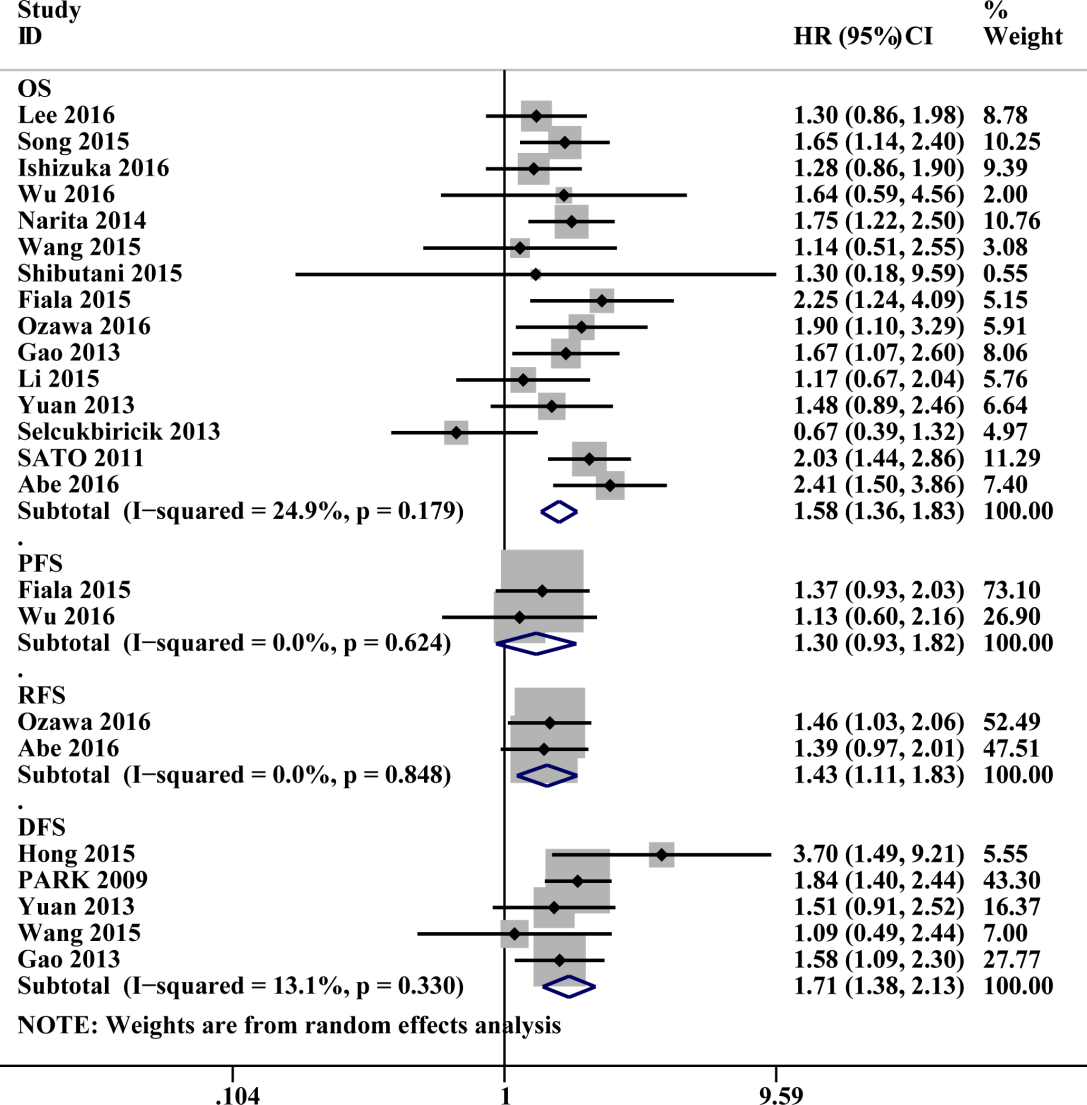
Forest plots of studies evaluating hazard ratios of pretreatment serum carbohydrate antigen 19–9 level (CA199) in patients with colorectal cancer (CRC). (1) Pretreatment serum CA199 level was associated with shorter overall survival (OS) in CRC; (2) Pretreatment serum CA199 level was associated with shorter disease-free survival, progression-free survival, recurrence-free survival in CRC.

Data collected from 15 studies involving 5163 patients with CRC were investigated to determine the association between pretreatment serum CA 19–9 level and OS. High pretreatment serum CA 19–9 levels were found to be associated with poor prognosis and low OS (HR: 1.58, 95% CI: 1.36–1.83, P<0.001), and there was no significant heterogeneity between these studies (*P* = 0.179, *I*^*2*^ = 24.9%; Fig 2).

#### 3.2.2. Disease-free survival

Data on DFS were provided by five studies involving 2694 patients. The data indicated an association between high pretreatment serum CA 19–9 levels and poor DFS (HR: 1.71, 95% CI: 1.38–2.13, *P*<0.001), and there was no heterogeneity between studies (*P* = 0.330, *I*^*2*^ = 13.1%; Fig 2).

#### 3.2.3. Progression-free survival and recurrence-free survival

Overall, data on PFS were available for 207 patients and showed that elevated pretreatment levels of serum CA 19–9 were associated with poor prognosis (HR: 1.30,95%CI:0.93–1.82, *P* = 0.121), we have got the same conclusion in RFS with 302 CRC patients (HR: 1.43, 95% CI: 1.11–1.83, *P* = 0.006). Subgroup analysis was not performed in view of the small number of RFS and PFS.

#### 3.2.4. Subgroup analysis

Subgroup analysis was used to evaluate the data pertaining to geographical area, distant metastasis, the type of analysis, source of HRs, sample size, and the treatment method. Heterogeneity occurred in the area subgroup and the data do not account for the small sample size. There was no significant difference between the rest subgroups (Table 2).

**Table 2:** Pooled hazard ratios (HRs) for OS according to subgroup analyses.

Variables	Outcome	Studies	Patients	HR (95% CI)	*P* value	Model	Heterogeneity	
							I^2^	*P*
**All**	OS	15	5163	1.58(1.36, 1.83)	<0.001	fixed	24.9%	0.179
	DFS	5	2694	1.71(1.38, 2.13)	<0.001	fixed	13.1%	0.330
**Area**								
Eastern	OS	13	4796	1.63 (1.42, 1.87)	<0.001	fixed	0.0%	0.579
	DFS	5	2694	1.71(1.38, 2.13)	<0.001	fixed	13.1%	0.330
Western	OS	2	367	1.23(0.38, 4.03)	0.773	random	87.1%	0.005
	DFS	0	0	-	-	-	-	-
Analysis type^*^								
Univariate	OS	4	606	1.50(1.15, 1.95)	<0.001	fixed	0.0%	0.865
Multivariate	OS	11	4557	1.58(1.30, 1.93)	<0.001	fixed	43.3%	0.062
	DFS	5	2694	1.71(1.38, 2.13)	<0.001	fixed	13.1%	0.330
Metastasis^*^								
Mixed	OS	11	2871	1.51(1.25, 1.83)	<0.001	fixed	34.9%	0.119
	DFS	3	1275	1.70(1.27,2.26)	0.004	fixed	36.2%	0.209
No	OS	4	2292	1.80(1.42, 2.28)	<0.001	fixed	0.0%	0.608
	DFS	2	1419	1.63 (1.06, 2.51)	0.026	fixed	31.2%	0.228
**HR obtain method**								
Reported in text	OS	14	5034	1.53(1.32, 1.77)	<0.001	fixed	16.0%	0.279
	DFS	4	1585	1.63(1.25, 2.12)	0.001	fixed	6.5%	0.370
Data-extrapolated	OS	1	129	2.41(1.50,3.86)	-	-	-	-
	DFS	1	1109	1.84(1.40,2.44)	-	-	-	-
Sample size^*^								
>200	OS	8	4247	1.46(1.16,1.84)	<0.001	fixed	39.9%	0.113
	DFS	4	2532	1.66(1.36,2.02)	<0.001	fixed	0.0%	0.622
<200	OS	7	916	1.69(1.39,2.04)	<0.001	fixed	5.9%	0.382
	DFS	1	162	3.699(1.486,9.209)	-	-	-	-
Treatment method^*^								
operate	OS	9	3300	1.66(1.40,1.96)	<0.001	fixed	2.3%	0.413
	DFS	5	2694	1.71(1.38, 2.13)	<0.001	fixed	13.1%	0.330
mixed	OS	6	1863	1.57(1.28,1.94)	<0.001	random	52.8%	0.048

Abbreviation: OS overall survival, DFS disease-free survival, PFS progression-free survival, RFS relapse-free survival, HR hazard ratio, CI confidence intervals.

*indicates that the difference was statistically significant

### 3.3. Sensitivity analyses

**Fig 3:**
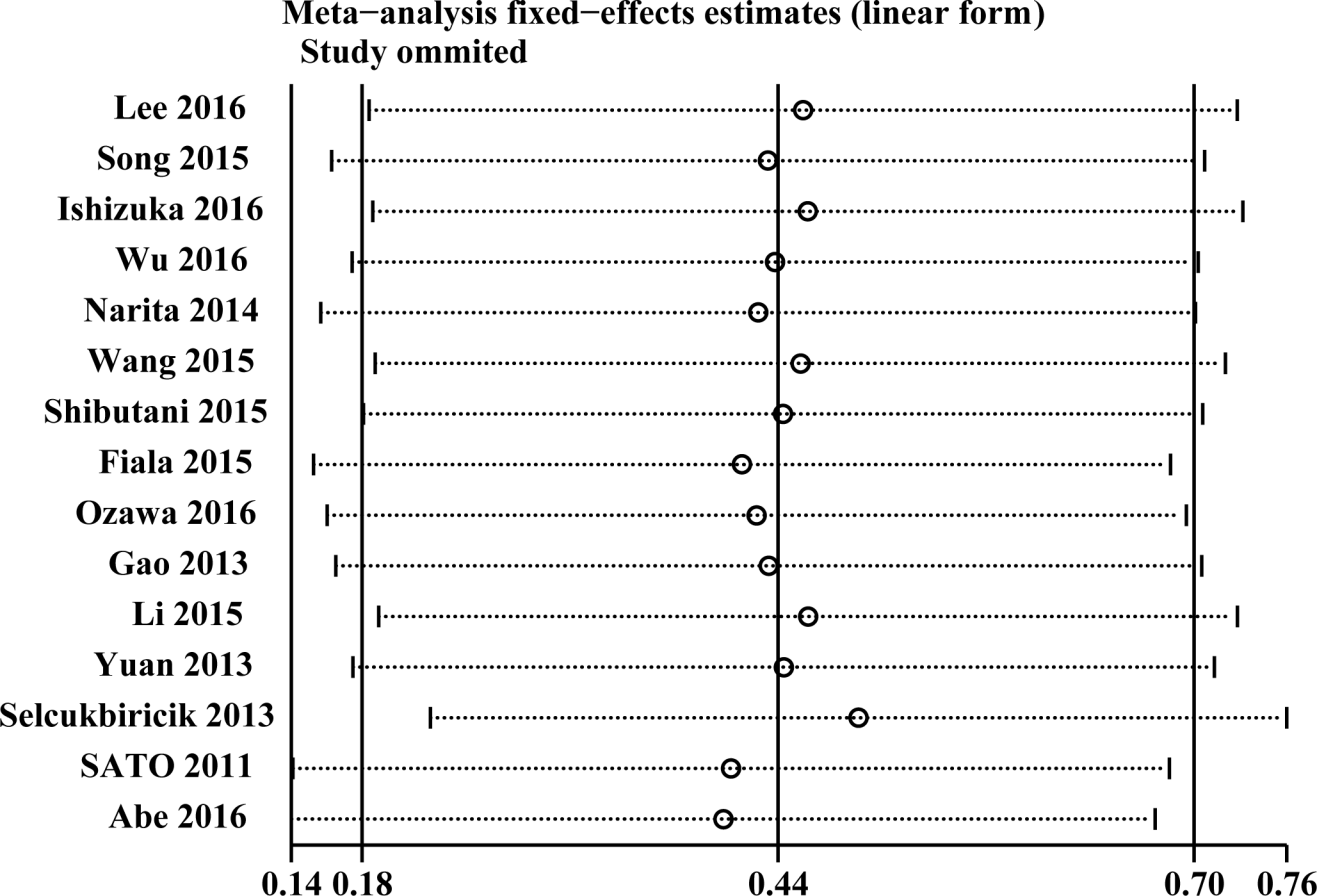
Sensitivity analyses for confirming robustness of OS by removing 1 study each time.

We did sensitivity analysis only for OS. We removed each of the articles to assess the impact of the individual data of each of these studies on the results of this study and found no significant change in the combinations of HRs and 95% CIs. This finding reflected the stability of the outcome (Fig 3).

### 3.4. Publication bias

**Fig 4:**
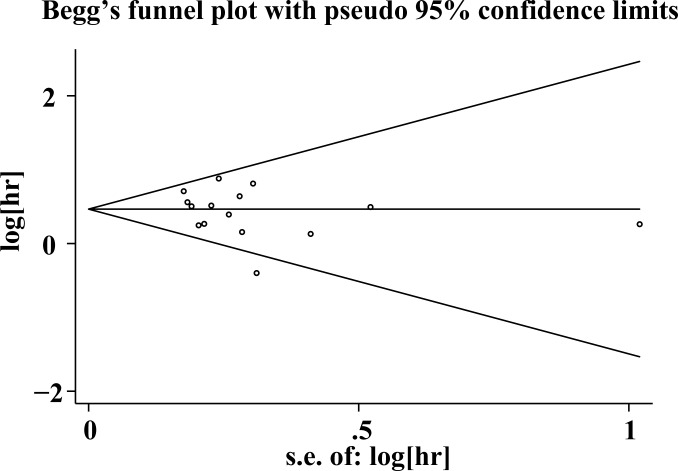
Funnel plots for the evaluation of potential publication bias of OS for CRC.

The publication bias in each of the included studies was calculated only for OS by funnel plots as well as Egger’s and Begg’s tests. The funnel plots were almost symmetrical (Fig 4). Furthermore, Egger’s and Begg’s test results revealed no significant publication bias in our study (OS: *P* = 0.347, 0.373).

## 4. Discussion

In this meta-analysis, we investigated the relationship between the pretreatment serum CA 19–9 levels and prognosis in 17 studies comprising 6434 patients with CRC. Our final results indicate a significant correlation between elevated pretreatment serum CA 19–9 levels and poor prognosis in CRC patients, with a combined HR of 1.58 (95% CI: 1.36–1.83, *P*<0.001), for OS, 1.71 (95% CI: 1.38–2.13, *P*<0.001for DFS, 1.30 (95% CI: 0.93–1.82) for RFS and 1.43 (95% CI: 1.11–1.83) for PFS. Furthermore, subgroup analyses were conducted according to the tumor site, occurrence of distant metastasis, type of analysis, method of calculating HR, sample size, and treatment. The results of analyses with all these subgroups revealed that a high pretreatment serum CA 19–9 level indicates poor prognosis, and further indicated that there was no significant difference among these different subgroups.

Although several studies have attested to the association between pretreatment serum CA 19–9 levels and the prognosis of CRC, the underlying reasons continue to remain elusive. CA 19–9 has been used in the diagnosis and prognosis of pancreatic cancer, CRC, gastric cancer, and other gastrointestinal tumors [32–34]. In CRC patients, the role of CA 19–9 remains controversial since it has a lower sensitivity as compared to CEA[35]. In the body, CA19-9 has been found to occur in salivary mucin and is distributed across the normal pancreas, gallbladder, liver, intestine, bile duct epithelium, etc. It is a cell surface glycoprotein and is involved in cellular adhesion, cancer cells expressing this protein may have greater metastatic and invasive potential [27]. Additionally, it has been reported to mediate the adhesion of tumor cells to the endothelial cells of blood vessels, thereby contributing to tumor metastasis [36]. Moreover, the presence of CA 19–9 correlate with the occurrence of tumor cell-induced platelet aggregation, which is an important process involved in the distant metastasis of CRC [37]. Furthermore, the possible involvement of CA 19–9 in tumor recurrence and survival may be attributed to differences in chemotherapy resistance or biological features of stage IV CRC, compared to other stages [17]. Such as it is reported that the baseline CA 19–9 level was an independent prognostic factor in metastatic CRC and also a predictive factor of bevacizumab efficacy, and the baseline CA 19–9 level correlated with the KRAS/BRAF mutation status [27]. This is also reported in another research [29]. CA 19–9 has also been reported to play a role in the occurrence of cancer invasion by enhancing cell adhesion and indirectly promoting angiogenesis [38]. Put together, these explanations, at least partly, explain the negative correlation of pretreatment serum CA 19–9 levels and prognosis.

This study has some limitations. First, our sample size was relatively small. Second, owing to the diversity of the cut-off values defined in each of these studies, we could not perform further subgroup analysis. Third, these included studies were heterogeneous due to differences in design, which also did not allow for the assessment of other clinical features.

## 5. Conclusion

Thus, our meta-analysis revealed an association between high pre-treatment serum CA 19–9 levels and poor survival in patients with CRC. Hence, serum CA 19–9 levels may be used as a marker to facilitate the treatment planning and prognostic evaluation of CRC patients. As a common tumor marker today, it has the advantage that it is cheaper, more convenient and more acceptable to patients. Further investigations are necessary to determine whether any association exists between serum CA 19–9 levels and survival.

## Supporting information

S1 File Plosone-meta-analysis-checklist. (DOCX)

S2 File PRISMA 2009 checklist. (DOC)

## Abbreviations

CA 19–9: Carbohydrate antigen 19–9
CRC: colorectal cancer
OS: overall survival
DFS: disease-free survival
PFS: progression-free survival
RFS: recurrence-free survival
HR: hazard ratio
CI: confidence interval.
